# Host inflammatory markers and MLVA profiling for risk assessment of severe pediatric *Mycoplasma pneumoniae* pneumonia

**DOI:** 10.3389/fcimb.2026.1828797

**Published:** 2026-07-24

**Authors:** Huan Xu, Jingmin Zhang, Shujuan Jiang, Jingxuan Xu, Xiaoqian Huang, Shuning Sun, Han Yan, Zhaoyong Lv, Daogang Qin, Jing Wang, Ting Wang

**Affiliations:** 1 Key Laboratory for Pediatrics of Integrated Traditional and Western Medicine, Liaocheng People’s Hospital, Liaocheng, Shandong, China; 2 Department of Pediatrics, Liaocheng People’s Hospital, Liaocheng, Shandong, China

**Keywords:** children, macrolide resistance, multilocus variable-number tandem repeat analysis (MLVA), *Mycoplasma pneumoniae* (MP), severe *Mycoplasma pneumoniae* pneumonia (SMPP)

## Abstract

**Background:**

*Mycoplasma pneumoniae* (MP) is a leading cause of community-acquired pneumonia in children (CAP). Rising macrolide resistance and variability in clinical severity have become growing concerns. This study aimed to describe the molecular epidemiology, resistance patterns, and risk factors for severe *M. pneumoniae* pneumonia (SMPP) in children from Liaocheng, Shandong Province, China.

**Methods:**

We retrospectively enrolled 421 children hospitalized with MP pneumonia (MPP) between January 2023 and December 2024 in this study. Clinical and laboratory data were collected. *M. pneumoniae*-DNA-positive specimens were analyzed by 23S rRNA gene sequencing to identify macrolide resistance mutations, and by Multilocus Variable-Number Tandem Repeat Analysis (MLVA) for molecular typing. Univariate and multivariate logistic regression analyses were used to identify risk factors for SMPP.

**Results:**

Of the 421 patients, 212 (50.4%) were diagnosed with SMPP. Children with SMPP were significantly older than those with general MPP (GMPP) (*P* = 0.001). Elevated D-dimer and lactate dehydrogenase (LDH) levels were identified as independent risk factors for SMPP. Among 148 successfully sequenced specimens, 144 (97.30%) carried macrolide resistance mutations, with A2063G being the most common (96.62%). MLVA typing identified 14 distinct subtypes, the most prevalent were M2-4-5-7-2, M5-4-5-7-2, M3-4-5-7-2, and M4-3-5-6-2. The subtype M4-3-5-6–2 was associated with a lower risk of SMPP (OR = 0.155, 95% CI: 0.037-0.654), while the subtype M3-4-5-7–2 carried the highest risk (OR = 14.0, *P* = 0.001).

**Conclusion:**

This study reveals a high rate of macrolide-resistant MP in Liaocheng, largely caused by the A2063G mutation. Elevated D-dimer and elevated LDH are independent predictors of SMPP. Certain MLVA subtypes are significantly associated with disease severity, suggesting they may help in risk assessment and clinical decision-making.

## 1 Introduction

*Mycoplasma pneumoniae* pneumonia (MPP) is a leading cause of community-acquired pneumonia (CAP) in children worldwide, and represents a significant burden on pediatric respiratory health (Cillóniz et al., 2016; Kutty et al., 2019). In China, MPP accounts for approximately 40% of pediatric CAP cases (Zhang et al., 2025; Zhu et al., 2026) and mostly affects children aged 5 years and older (Jiang et al., 2023; Sun Q. et al., 2025; Varela et al., 2022). While most MPP infections are self-limiting, delayed diagnosis and inadequate treatment may lead to severe MPP (SMPP). This severe phenotype is associated with life-threatening complications, including extensive pulmonary consolidation, necrotizing pneumonia, and pleural effusion (Lee et al., 2020; Zhu et al., 2026).

The widespread emergence and transmission of macrolide-resistant *M. pneumoniae* (MRMP) has become a major clinical challenge. Initially identified in Japan in 2000, MRMP has rapidly disseminated across the globe over the past two decades (Kim et al., 2022; Jiang et al., 2024). In China, resistance rates are particularly high, exceeding 93.02% nationally (Sun Y. et al., 2025) and even 100% in some regions (Jia et al., 2024). Patients infected with MRMP tend to have more severe symptoms, longer fevers, and longer hospital stays (Xu et al., 2024; Wang et al., 2024). These poor outcomes highlight the urgent need for early resistance testing to guide better antibiotic choices for individual patients.

*M. pneumoniae* exhibits distinct periodic epidemic patterns with large-scale outbreaks recurring every 1 to 7 years and persisting 1 to 2 years (Chen et al., 2024; Yan et al., 2024). This recurring epidemiological feature highlights the importance of continuous molecular surveillance to monitor circulating strain profiles and evolutionary dynamics. Multiple-locus variable-number tandem-repeat analysis (MLVA) is a high-resolution genotyping tool that enables the discrimination of *M. pneumoniae* strains and facilitates the monitoring of epidemiological trends and transmission dynamics (Dégrange et al., 2009; Rivaya et al., 2020; Chen et al., 2024).

With this in mind, this study aimed to identify clinical and laboratory indicators capable of predicting SMPP in pediatric patients. Additionally, we conducted the first regional investigation in Liaocheng, Shandong Province, integrating macrolide resistance mutation detection and MLVA genotyping to characterize local circulating *Mycoplasma pneumoniae* strains. This comprehensive strategy was performed to clarify the molecular epidemiological profiles of endemic strains and explore the potential association between specific MLVA genotypes and MPP disease severity.

## 2 Methods

### 2.1 Patients and specimens

This study aimed to identify risk factors for SMPP. Due to an imbalanced distribution of SMPP and general MPP (GMPP) cases in the original cohort, a retrospective case-control design was adopted. To enhance statistical power for comparative analyses, all SMPP patients were included, and an equivalent number of GMPP patients were randomly selected for matching. The final study population comprised 421 hospitalized children with MPP who were admitted to Liaocheng People’s Hospital between January 1, 2023, and December 31, 2024.

All patients were diagnosed and treated according to the Evidence-based guideline for the diagnosis and treatment of *Mycoplasma pneumoniae* pneumonia in children (2023 Edition) (Subspecialty Group of Respiratory, the Society of Pediatrics, 2025). This guideline provides a two-step framework for case identification and severity classification. Severity of MPP was determined based on specified clinical indicators. A diagnosis of SMPP was made when patients presented with at least one of the following: prolonged or high-grade fever, respiratory distress, extrapulmonary complications, or resting hypoxemia with room-air SpO_2_ ≤ 93%. Radiographic findings indicative of severe disease included multilobar consolidation, pleural effusion, extensive pulmonary infiltrates, or significant radiological progression within 48 hours. A pronounced systemic inflammatory response-characterized by markedly elevated levels of C-reactive protein (CRP), lactate dehydrogenase (LDH), or D-dimer was also considered a marker of severity. Patients diagnosed with MPP who did not meet any of these criteria were classified into the GMPP group. All clinical records and laboratory results were systematically collected and recorded.

Ethical approval was obtained from the Ethics Committee of Liaocheng People’s Hospital. Individual informed consent was waived, as the DNA samples analyzed were de-identified residual specimens collected during routine clinical care.

Upon admission, nasopharyngeal swabs were collected from all patients, placed in 2 mL of normal saline, and stored at -80 °C. In addition, bronchoalveolar lavage fluid (BALF) was collected from patients who underwent clinically indicated bronchoscopy and was stored under the same conditions for subsequent analyses.

### 2.2 Nucleic acid extraction and detection of *M. pneumoniae*

Between January 2023 and December 2024, 5, 122 clinical specimens were collected from patients with suspected MPP. DNA was extracted from all samples, and *M. pneumoniae* DNA was detected by real-time PCR using the Diagnostic Kit for *Mycoplasma pneumoniae* DNA (PCR-Fluorescence Probing, Daan Gene Co., Ltd.) according to the manufacturer’s instructions. PCR amplification and signal detection were carried out on ABI Prism 7500 Real-Time PCR System. Specimens that tested positive for *M. pneumoniae* DNA were stored at -80 °C for subsequent 23S rRNA sequencing and MLVA typing.

### 2.3 Detection of macrolide resistance-associated mutations in the 23S rRNA domain V

Macrolide resistance was assessed in all 421 enrolled patients by amplifying and sequencing domain V of the 23S rRNA gene, targeting point mutations at positions A2054, A2058, A2063, A2064, A2067, and A2617. This was a genotypic analysis only; phenotypic antimicrobial susceptibility testing was not performed, as no viable isolates were available.

All primer pairs used in this assay were synthesized by Sangon Biotech (Shanghai, China), with the forward sequences 5′-GCAGTG AAG AA CGAGGGG-3′and reverse sequence 5′-GTCCTCGC TTCGGTCCTCTCG-3′.Target gene amplification was performed using Premix Taq™ Hot Start Version (Takara Bio, R028A) with a standardized thermal cycling protocol: initial denaturation at 95 °C for l0 min; followed by 40 cycles of 95 °C for 45 s, 57 °C for 60 s, and 72 °C for 60 s, and a final extension step at 72 °C for l0 min. After purification, all PCR products were submitted to Sangon Biotech for Sanger sequencing. Obtained sequences were aligned with the reference strain *Mycoplasma pneumoniae M129* (GenBank accession no. NC_000912.1) using BioEdit software to screen for resistance-related gene mutations.

### 2.4 MLVA typing

MLVA genotyping was performed directly on the DNA extracts without bacterial isolation or cultivation. MLVA typing of *M. pneumoniae-*positive DNA specimens was performed as described previously (Dégrange et al., 2009; Dumke and Jacobs, 2011). MLVA profiling was attempted on the same specimens that achieved successful 23S rRNA gene sequencing. Briefly, five variable-number tandem-repeat (VNTR) loci (Mpn1, Mpn13, Mpn14, Mpn15, and Mpn16) were amplified by PCR using locus-specific primers. All amplified products were purified and subjected to Sanger sequencing at Sangon Biotech (Shanghai, China). The repeat copy number at each VNTR locus was determined by sequence alignment with the reference strain *Mycoplasma pneumoniae M129* strain. Final MLVA types were defined based on the combination of repeat numbers across all five loci, presented in the standard format Mpn1-Mpn13-Mpn14-Mpn15-Mpn16.

### 2.5 Statistical analysis

Statistical analyses were performed using IBM SPSS Statistics 25. The normality of continuous variables was assessed with appropriate tests. Normally distributed data are presented as mean ± standard deviation (x̄ ± s) and were compared between groups using the independent samples t-test. Non-normally distributed data are expressed as median (interquartile range) [M (QL, QU)] and were analyzed with the Mann–Whitney U test. Post-hoc pairwise comparisons were adjusted using the Bonferroni correction. Categorical variables are summarized as number (percentage) and were compared using the Pearson χ² test when all expected frequencies were ≥ 5; the continuity-corrected χ² test when any expected frequency was < 5 but ≥ 1; and Fisher’s exact test when any expected frequency was < 1 or 2×T < 5.

To identify independent risk factors for SMPP, variables showing significant differences in univariate analyses were entered into a binary logistic regression model. The association between MLVA subtypes and the incidence of SMPP was first examined using the row × column χ² test, followed by binary logistic regression. A two-tailed *P-*value < 0.05 was considered statistically significant. For pairwise MLVA subtypes comparisons, Bonferroni correction was applied (four comparisons per model; adjusted α = 0.05/4 = 0.0125).

## 3 Results

### 3.1 General data

A total of 421 children diagnosed with MPP were included in this study, including 216 males (51.3%) and 205 females (48.7%), aged 0.3 to 14 years. The median age of the participants was 6 years (interquartile range (IQR): 4–7 years). The 3–6 years age group accounted for the largest proportion of cases (n = 195, 46.32%), followed by children aged over 6 years (n = 153, 36.34%) and those aged 3 years or younger (n = 73, 17.34%).

According to disease severity, all patients were divided into GMPP group (n = 209) and SMPP group (n = 212). The median age at onset was significantly higher in the SMPP group compared to the GMPP group (*P* = 0.001). Subsequent age-stratified analysis with a Bonferroni-adjusted significance threshold (*P* < 0.0167) indicated that GMPP group had a higher proportion of children ≤ 3 years subgroup (22.0% vs. 12.7%), whereas SMPP group was predominant in children aged > 6 years (43.4% vs. 28.7%). No significant difference in disease severity distribution was observed in the 3–6 years age group (49.3% vs. 43.4%) (**Table 1**).

**Table 1:** Comparison of clinical characteristics and laboratory findings between GMPP and SMPP.

Variables		GMPP (n = 209)	SMPP (n = 212)	Z/X^2^	*P*
General information	Age (y), Median (IQR)	6 (4, 7)	6 (4, 7)	-3.463	0.001
	≤3y	46 (22.0)	27 (12.7)		0.012
	3y <age ≤6y	103 (49.3)	92 (43.4)		0.226
	>6y	60 (28.7)	93 (43.4)		0.001
Clinical presentation	Sex ratio (M/F)	114/95	102/110	1.743	0.187
	Cough	208 (99.5)	212 (100.0)	–	0.496
	Expectoration	191 (91.4)	203 (95.6)	3.344	0.067
	Fever days, Median (IQR)	5 (3, 7)	6 (4, 7)	-4.363	< 0.001
	Hot peak (°C), Median (IQR)	39.0 (38.5, 39.5)	39.2 (38.9, 39.6)	-3.929	< 0.001
	Dyspnea	3 (1.4)	9 (4.2)	3.030	0.082
	Coinfection	73 (34.9)	55^c^ (25.9)	2.994	0.084
	Virus	35	18		> 0.05
	Bacteria	38	37		> 0.05
Laboratory Examination	D-dimer elevation	7 (3.3)	69 (32.5)	60.651	< 0.001
	WBC (10^9^/L), Median (IQR)	8.00 (6.51, 10.25)	8.26 (6.85, 10.42)	-0.843	0.405
	N%	60.5 (50.0, 69.0)	66.1 (57.3, 74.1)	-4.638	< 0.001
	L%	28.2 (21.3, 38.1)	24.1 (17.8, 31.5)	-4.237	< 0.001
	NLR	2.11 (1.25, 3.09)	2.74 (1.81, 4.17)	-4.603	< 0.001
	MLR	0.29 (0.21, 0.38)	0.31 (0.23, 0.44)	-1.396	0.163
	PLR	117.22 (82.05, 168.53)	138.32 (105.21, 204.79)	-3.953	< 0.001
	M%	8.5 (6.4, 10.4)	7.5 (5.4, 10.3)	-2.696	0.007
	PLT (10^9^/L), Median (IQR)	267.0 (226.0, 347.0)	283.0 (233.0, 347.0)	-1.098	0.272
	MPV (fL), Median (IQR)	9.3 (8.7, 9.7)	9.3 (8.7, 10.0)	-0.587	0.557
	MPVLR	3.92 (2.71, 5.71)	4.64 (3.49, 6.49)	-3.483	< 0.001
	CRP (mg/L), Median (IQR)	10.96 (4.13, 24.58)	17.75 (7.53, 36.61)	-3.902	< 0.001
	A/G	1.50 (1.35, 1.68)	1.46 (1.24, 1.62)	-3.242	0.001
	LDH (U/L), Median (IQR)	270 (238, 320)	308 (258, 417)	-5.298	< 0.001

^a GMPP, general Mycoplasma pneumoniae pneumonia; SMPP, severe Mycoplasma pneumoniae pneumonia; IQR, interquartile range; WBC, white blood cell count; N%, percentage of neutrophils; L%, percentage of lymphocytes; NLR, neutrophil-to-lymphocyte ratio; MLR, monocyte-to-lymphocyte ratio; PLR, platelet-to-lymphocyte ratio; M%, percentage of monocytes; PLT, platelet count; MPV, mean platelet volume; MPVLR, mean platelet volume to platelet count ratio; CRP, C-reactive protein; A/G, albumin to globulin ratio; LDH, lactate dehydrogenase.

^b Data are presented as median (IQR) or n (%)

^c Data are presented as the number of patients with co-infection included in the statistical analysis. Fourteen SMPP cases positive only for *Streptococcus viridans* were excluded.

### 3.2 Clinical manifestations and laboratory findings

Among the 421 children with MPP, fever was observed in 395 cases (93.8%), with peak body temperature ranging from 37.5 °C to 41 °C. The duration of fever prior to hospitalization varied from 0 to 47 days. Cough was reported in 420 cases (99.8%), with expectoration in 394 cases (93.6%). Dyspnea was observed in 12 patients (2.9%).

Compared with the GMPP group, children in the SMPP group had significantly longer fever duration before admission (median 6 days vs. 5 days; *P* < 0.001) and higher peak body temperatures (median 39.2 °C vs. 39.0 °C; *P* < 0.001). No significant differences were found between the two groups in the incidence of cough (100.0% vs. 99.5%; *P* = 0.496), expectoration (95.6% vs. 91.4%; *P* = 0.067), and dyspnea (4.2% vs. 1.4%; *P* = 0.082) (**Table 1**).

Overall, 142 patients (33.1%, 142/421) presented with co-infections. Prior to comparing co-infection rates across the two severity groups, we ruled out 14 SMPP cases. These patients harbored only *Streptococcus viridans*, a typical respiratory commensal, and were not given targeted antimicrobial treatment. Consequently, 73 patients in the GMPP group and 55 patients in the SMPP group were included in the final statistical comparison (**Table 1**). There were no statistically significant difference between groups (*P* = 0.084). When co-infecting pathogens were categorized as bacterial or viral, the distribution remained comparable between groups (adjusted residual = ± 1.7) (**Table 1**).

In terms of laboratory indices, SMPP patients presented significantly higher levels of neutrophil percentage (Neu%; median: 66.1% vs. 60.5%; *P* < 0.001), neutrophil-to-lymphocyte ratio (NLR; median: 2.74 vs. 2.11; *P* < 0.001), platelet-to-lymphocyte ratio (PLR; median: 138.32 vs. 117.22; *P* < 0.001), mean platelet volume-to-lymphocyte ratio (MPVLR; median: 4.64 vs. 3.92; *P* < 0.001), C-reactive protein (CRP; median: 17.75 vs. 10.96 mg/L; *P* < 0.001), D-dimer elevation (32.5% vs. 3.3%; *P* < 0.001), and LDH (median: 308 vs. 270 U/L; *P* < 0.001). In contrast, patients with SMPP showed significantly lower lymphocyte percentage (Lym%; median: 24.1% vs. 28.2%; *P* < 0.001), monocyte percentage (M%; median: 7.5% vs. 8.5%; *P* = 0.007), and the albumin-to-globulin ratio (A/G; median: 1.46 vs. 1.50; *P* = 0.001). No significant intergroup differences were found in white blood cell count (WBC), monocyte-lymphocyte ratio (MLR), platelet count (PLT), or mean platelet volume (MPV) (all *P* > 0.05) (**Table 1**).

### 3.3 Factors associated with SMPP: univariate and multivariate logistic regression analysis

We first performed univariate logistic regression and identified 13 potential risk factors for SMPP. All these factors were subsequently included in the multivariate binary logistic regression model. The results demonstrated that elevated D-dimer (OR = 8.301, 95% CI 6.327-10.890, *P* < 0.001) and raised lactate dehydrogenase (LDH; OR = 1.006, 95% CI 1.002-1.010, *P* = 0.003) were independent risk factors for SMPP (**Table 2**).

**Table 2:** Univariate and multivariate logistic regression analysis of factors associated with SMPP in children.

Variables	Univariate logistic regression		Multivariate logistic regression	
	OR (95% CI)P	*P*	OR (95% CI)P	
Age (y)	1.167 (1.073-1.269)	< 0.001	1.148 (0.983-1.341)	0.081
Fever days	1.086 (1.024-1.152)	0.006	0.981 (0.918-1.049)	0.577
Hot peak (°C)	1.874 (1.429-2.457)	< 0.001	1.473 (0.991-2.190)	0.882
D-dimer elevation	13.924 (10.790-17.969)	< 0.001	8.301 (6.327-10.890)	< 0.001
N%	1.035 (1.020-1.051)	< 0.001	0.995 (0.931-1.064)	0.882
L%	0.964 (1.948-0.981)	< 0.001	0.980 (0.914-1.051)	0.568
M%	0.936 (0.552-0.992)	0.027	1.025 (0.929-1.131)	0.622
NLR	1.125 (1.090-1.354)	< 0.001	1.100 (0.864-1.401)	0.440
PLTLR	1.005 (1.002-1.007)	< 0.001	1.003 (0.997-1.008)	0.382
MPVLR	1.130 (1.051-1.227)	0.001	0.871 (0.723-1.051)	0.149
CRP (mg/L)	1.015 (1.007-1.024)	< 0.001	1.000 (0.989-1.011)	0.989
A/G	0.258 (0.118-0.563)	0.001	1.015 (0.283-3.649)	0.981
LDH (U/L)	1.009 (1.006-1.012)	< 0.001	1.006 (1.002-1.010)	0.003

OR, odds ratio; CI, confidence interval; Other abbreviations as in **Table 1**.

### 3.4 Prevalence and molecular characterization of macrolide resistance in MPP

Of the 5, 122 clinical specimens tested, 685 (13.37%) were positive for *M. pneumoniae* DNA. Additionally, among the 421 enrolled patients, 218 had DNA of sufficient quality for 23S rRNA amplification and macrolide resistance genotyping. Usable sequencing data were obtained for 148 specimens, giving a success rate of 67.89% (148/218). Of these 148 specimens, 144 (97.30%) carried mutations in the 23S rRNA gene. The A2063G transition was the predominant mutation, detected in 143 specimens (96.62%), where the A2064G mutation was found in one specimen (0.68%). Four specimens (2.70%) showed no mutations at the targeted loci.

### 3.5 Association between 23S rRNA mutations and clinical severity

The 148 patients with successful sequencing were divided into GMPP (n = 52) and SMPP (n = 96) groups according to clinical severity. The A2063G or A2064G resistance mutations were found in 51 of 52 GMPP patients (98.07%) and 93 of 96 SMPP patients (96.88%), with no statistically significant difference between the two groups (*P* = 1.000).

### 3.6 Molecular typing and genetic diversity of *M. pneumoniae* strains

**Figure 1:**
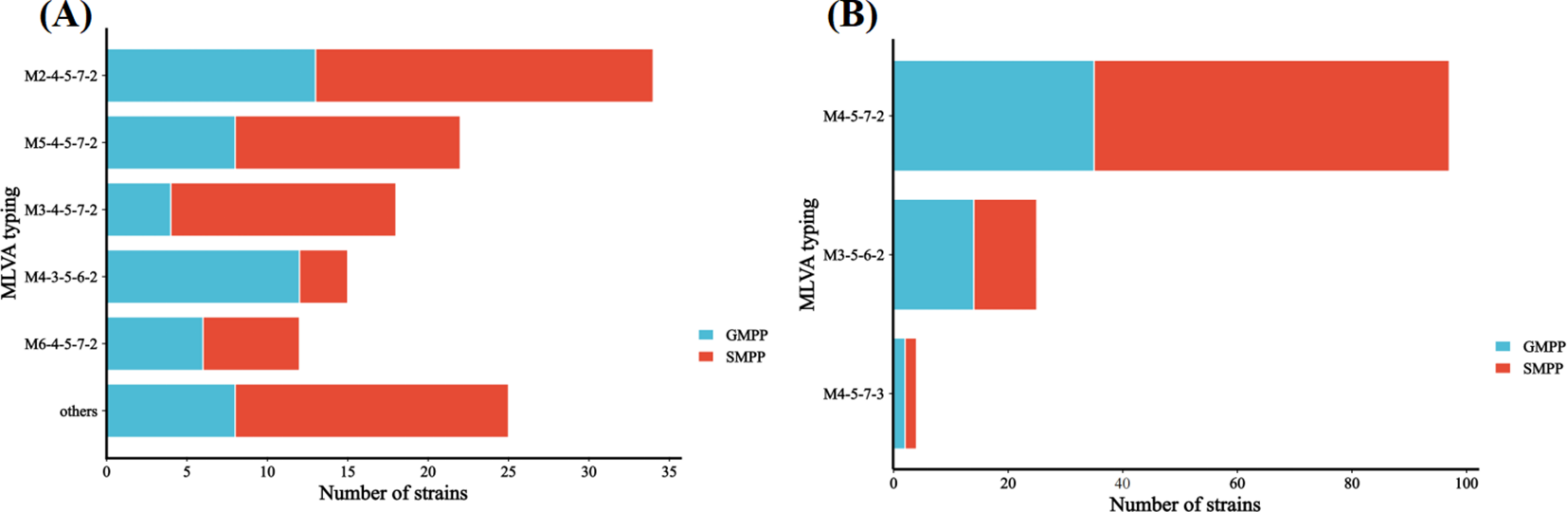
Distribution of Major MLVA typing and Clinical Groups in 126 Pediatric MPP Patients. **(A)** Five-locus Scheme (Mpn1, Mpn13-16). **(B)** Four-locus Scheme (Mpn13-16).

Of the 148 specimens successfully sequenced for the 23S rRNA gene, 126 (85.1%) gave complete MLVA genotyping results. Using the five loci (Mpn1, 13-16) scheme, we identified 14 distinct MLVA genotypes. Four subtypes were most common: M2-4-5-7-2 (n = 34, 26.98%), M5-4-5-7-2 (n = 22, 17.46%), M3-4-5-7-2 (n = 18, 14.29%), and M4-3-5-6-2 (n = 15, 11.90%) (**Figure 1**). Notably, one strain exhibited heterozygosity at the Mpn1 locus, with short tandem repeat (STR) copy numbers of 3 and 4. This strain was assigned the heterozygous genotype M3/4-4-5-7-2.

When we excluded the Mpn1 locus and used a four loci (Mpn13-16) scheme, the typing resolution was dropped considerably, and the isolates fell into just three genotypes: M4-5-7-2 (n = 97), M3-5-6-2 (n = 25), and M4-5-7-3 (n = 4). Importantly, the first three predominant five loci subtypes (M2-4-5-7-2, M5-4-5-7-2, and M3-4-5-7-2) all mapped to the same four loci genotype M4-5-7-2.

The distribution of the major MLVA subtypes differed between the two clinical groups (**Figure 1**). Subtypes M2-4-5-7-2 (28.0%), M5-4-5-7-2 (18.7%), and M3-4-5-7-2 (18.7%) were more common in SMPP than in GMPP patients. In contrast, subtype M4-3-5-6-2 (23.5%) was predominantly found in the GMPP group. The full distribution of all subtypes across the two groups is shown in **Table 3**.

**Table 3:** MLVA profiles in 126 *M. pneumoniae* isolates from children with MPP.

MLVA profile		All n = 126 N (%)	GMPP n = 51 N (%)	SMPP n = 75 N (%)
four loci	five loci			
4-5-7-2		97 (77.0)	35 (68.6)	62 (82.7)
	2-4-5-7-2	34 (27.0)	13 (25.5)	21 (28.0)
	5-4-5-7-2	22 (17.5)	8 (15.7)	14 (18.7)
	3-4-5-7-2	18 (14.3)	4 (7.8)	14 (18.7)
	6-4-5-7-2	12 (9.5)	6 (11.8)	6 (8.0)
	7-4-5-7-2	5 (4.0)	1 (2.0)	4 (5.3)
	4-4-5-7-2	5 (4.0)	2 (3.9)	3 (4.0)
	3/4-4-5-7-2	1 (0.8)	1 (2.0)	0 (0)
3-5-6-2		25 (19.8)	14 (27.5)	11 (14.7)
	4-3-5-6-2	15 (11.9)	12 (23.5)	3 (4.0)
	5-3-5-6-2	5 (4.0)	1 (2.0)	4 (5.3)
	2-3-5-6-2	4 (3.2)	1 (2.0)	3 (4.0)
	6-3-5-6-2	1 (0.8)	0 (0)	1 (1.3)
4-5-7-3		4 (3.2)	2 (3.9)	2 (2.7)
	3-4-5-7-3	1 (0.8)	0 (0)	1 (1.3)
	4-4-5-7-3	2 (1.6)	1 (2.0)	1 (1.3)
	7-4-5-7-3	1 (0.8)	1 (2.0)	0 (0)

Data are presented as the number of patients (percentage). Percentages were calculated based on the total cohort (n = 126) for the “All” column, and respective group totals (n = 51 for GMPP, n = 75 for SMPP) for subgroup columns.

### 3.7 Association between MLVA subtypes and macrolide resistance

Among the 126 isolates with complete MLVA typing, macrolide resistance patterns varied by subtype. All 97 M4-5-7–2 isolates (100%) carried 23S rRNA mutations. Of the 25 M3-5-6–2 isolates, 24 (96.0%) had detectable 23S rRNA mutations, while 1 (4.0%) showed no mutation. In contrast, only 1 of the 4 M4-5-7–3 isolates (25.0%) carried a 23S rRNA mutation; the other 3 (75.0%) were susceptible.

### 3.8 Association between MLVA subtypes and disease severity

M2-4-5-7-2, M5-4-5-7-2, M3-4-5-7-2, and M4-3-5-6–2 were identified as the predominant MLVA subtypes. The remaining subtypes had too few cases to analyze individually, so they were grouped together as “others” for statistical analysis. A 5 × 2 contingency table chi-square test revealed a significant association between MLVA subtype and disease severity (χ² = 12.547, *P* = 0.014) (**Table 4**).

**Table 4:** Association between MLVA subtypes and clinical severity in pediatric MPP.

Disease	Cases	M4-3-5-6-2	M5-4-5-7-2	M3-4-5-7-2	M2-4-5-7-2	Others
GMPP	51	12	8	4	13	14
SMPP	75	3	14	14	21	23
X^2^	12.547					
*P*	0.014					

To further quantify the risk of severe disease associated with each subtype, we performed binary logistic regression using two different reference strategies. In Model 1, we used the most frequent subtype M2-4-5-7–2 as the reference. Compared to this baseline, subtype M4-3-5-6–2 was significantly lower risk of SMPP (OR = 0.155, 95% CI: 0.037–0.654, *P* = 0.011). No significant differences were observed for subtypes M5-4-5-7–2 and M3-4-5-7–2 compared to subtype M2-4-5-7-2 (**Table 5**). Because the SMPP incidence in M2-4-5-7–2 was fairly high (61.7%), using it as reference may introduce baseline confounding. Therefore, in Model 2, we selected subtype M4-3-5-6-2 (lowest SMPP incidence) as the new reference. Under this model, subtype M3-4-5-7–2 showed the strongest association with SMPP (OR = 14.000, *P* = 0.002), followed by subtypes M5-4-5-7-2 (OR = 7.000, *P* = 0.013), and M2-4-5-7-2 (OR = 6.642, *P* = 0.011) (**Table 5**). To account for multiple comparisons, we applied a Bonferroni correction within each model. With four comparisons per model, the adjusted significance threshold was set at *P* < 0.0125. After correction, the protective effect of M4-3-5-6–2 in Model 1 (*P* = 0.011) remained significant. In Model 2, the elevated risks for M3-4-5-7-2 (*P* = 0.002) and M2-4-5-7-2 (*P* = 0.011) stayed significant, while M5-4-5-7-2 (*P* = 0.013) fell just short of the adjusted threshold.

**Table 5:** Association between MLVA subtypes and risk of severe MPP (SMPP) using different reference subtypes.

MLVA type	OR (95% CI)	*P*
Subtype M2-4-5-7–2 as reference		
M4-3-5-6-2	0.155 (0.037-0.654)	0.011
M3-4-5-7-2	2.167 (0.585-8.021)	0.247
M5-4-5-7-2	1.083 (0.357-3.289)	0.888
others	1.017 (0.390-2.654)	0.973
Subtype M4-3-5-6–2 as reference		
M3-4-5-7-2	14.000 (2.599-75.408)	0.002
M5-4-5-7-2	7.000 (1.509-32.478)	0.013
M2-4-5-7-2	6.642 (1.528-27.324)	0.011
others	6.571 (1.574-27.432)	0.010

*P* values were adjusted for multiple comparisons within each model using the Bonferroni method (adjusted α = 0.05/4 = 0.0125). Only P < 0.0125 was considered statistically significant.

When the four loci scheme (Mpn13–16) was applied, the isolates were grouped into three genotypes: M4-5-7-2, M3-5-6-2, and M4-5-7-3. A chi-square test showed no significant association between these genotypes and disease severity (χ² = 3.428, *P* = 0.180).

## 4 Discussion

This study provides the first systematic report on the epidemiological and molecular characteristics of *M. pneumoniae* infection in Liaocheng, Shandong Province, China, from 2023 to 2024, and identifies key risk factors for SMPP. We report an exceptionally high local prevalence of macrolide resistance. MLVA genotyping revealed four dominant circulating strains, among which subtype M4-3-5-6–2 demonstrated a protective effect against SMPP, whereas subtype M3-4-5-7–2 was associated with the highest risk. These findings establish a critical baseline for local surveillance and highlight the potential of molecular typing in clinical risk assessment.

### 4.1 Age−related distribution and severity in pediatric MPP

We found that SMPP was significantly more common in older children. This observation aligns with the concept of an age-dependent, exaggerated host inflammatory response. School-aged children typically mount a vigorous immune response to MP, which can trigger marked pulmonary inflammatory infiltration and excessive cytokine production. This exaggerated host reaction ultimately increases the likelihood of severe pulmonary injury and necrotizing pneumonia (Yang et al., 2021; Zhang et al., 2024a; Li et al., 2024). This finding is consistent with prior reports that refractory cases tend to occur in older children (Huang et al., 2021). By comparison, the clinical implications of immune immaturity in younger children remain inconclusive, as existing literature presents conflicting findings. Immature immune defenses may render young children more susceptible to severe infection in certain contexts (Zhang et al., 2024b), yet a blunted inflammatory response can also restrict immune-mediated lung damage and mitigate disease severity (Yang et al., 2021). Collectively, our data strengthen the evidence that advanced age serves as a critical epidemiological risk factor for SMPP. Furthermore, our clinical phenotypic observations help contextualize ongoing debates surrounding heterogeneous age-related immune responses and their impact on MPP severity.

### 4.2 Coinfection profiles and their association with disease severity in pediatric MPP

A central finding of our study was that 30.4% of children had coinfections, *Streptococcus pneumoniae* and rhinovirus were the most prevalent pathogens in both the GMPP and SMPP groups, which consistent with previous reports (Zhang et al., 2018; Jia et al., 2024; Li et al., 2024; Yuan et al., 2025). However, no statistically significant difference in overall bacterial or viral coinfection rates was observed between the two groups. This result contrasts with some reports associating higher co-infection rates with SMPP (Li et al., 2024; Yang et al., 2024). Instead, our results support the alternative view that the clinical relevance of coinfection is highly pathogen-specific. Severe disease complications in MPP are more reliably attributed to certain virulent pathogens, such as adenovirus and Epstein–Barr virus (EBV), rather than coinfection status itself (Gao et al., 2020; Yuan et al., 2025). Accordingly, our data indicate that the type of secondary pathogen, rather than the simple presence of coinfection, predominantly determines disease progression. This observation emphasizes the clinical value of precise pathogen detection for individualized risk assessment and targeted treatment of pediatric MPP.

### 4.3 Hematological inflammatory markers in GMPP and SMPP

Routine blood indices, including NLR, PLR, MLR, and MPVLR, are readily accessible markers of systemic inflammation and have been investigated as predictors of SMPP (Li D. et al., 2023; Ding and Jiang, 2025). While several routine hematological inflammatory markers (e.g., NLR, PLR) differed between groups in univariate analysis, none were retained as independent risk factors for SMPP in our multivariate model. This suggests that the predictive value of these readily accessible indices may be confounded in clinical practice by variables such as patient age and the timing of blood sampling relative to disease onset.

In contrast, elevated levels of LDH and D-dimer were independent predictors of SMPP in the present cohort. This strongly reinforces their established pathophysiological roles, directly linking markers of cellular injury (LDH) and a prothrombotic state (D-dimer) to disease severity, as reported previously (Qiu et al., 2022; Wang S. et al., 2023; Ding and Jiang, 2025). These results prioritize LDH and D-dimer as more reliable biomarkers for severity assessment in this context.

### 4.4 Global epidemiology and molecular basis of macrolide−resistant *M. pneumoniae*

Our study confirms an exceptionally high prevalence of MRMP in this region (97.30%), predominantly mediated by the A2063G mutation. This finding is consistent with the alarming trend of increasing resistance reported across China in recent years (Chen et al., 2024; Jia et al., 2024) and starkly contrasts with lower resistance rates in Europe and North America (Loconsole et al., 2021; Chen et al., 2024). Despite high genotypic resistance, azithromycin still works well in many pediatric patients, especially those with mild disease. This may be due to its favorable pharmacokinetics, immunomodulatory properties, and the self-limiting course of *M. pneumoniae* infection (Zheng et al., 2018; Jia et al., 2024; Zhang et al., 2024). Treatment should be individualized based on disease severity and clinical response, with early intervention within 5–10 days of fever onset being especially important. These findings underscore the value of early detection of resistance mutations to guide timely and appropriate therapy.

### 4.5 Molecular epidemiology and genotype distribution

Since late 2023, widespread *M. pneumoniae* outbreaks in China have been linked to both post-pandemic immune debt and shifts in circulating genotypes (Jia et al., 2024; Gong et al., 2024; Jiao et al., 2025; Wang et al., 2021; Li T. et al., 2023; Chen et al., 2024). Before and after the outbreak of the COVID-19 pandemic, the MLVA genotype M4-5-7–2 has been dominant in East Asia and increased in prevalence during the pandemic, while genotype 3-5-6–2 declined (Jiao et al., 2025). In China, the prevalence of M4-5-7–2 has gone up and down over time, recently rebounding back to 73.7% in 2023 (Jia et al., 2024; Chen et al., 2024). Genomic analyses indicate that the M4−5−7−2 subtype carries certain adaptive genetic features that may give it a transmission advantage, though this remains speculative and needs experimental confirmation (Jiao et al., 2025).

Our five loci MLVA scheme identified 14 distinct genotypes, with M2−4−5−7−2, M5−4−5−7−2, M3−4−5−7−2, and M4−3−5−6−2 being the most common. Under a four loci scheme, most isolates clustered into the M4-5-7–2 group, consistent with recent national surveillance and reflecting the current genotype distribution in this region (Lu et al., 2020; Chen et al., 2024). The fact that several five loci subtypes map to a single four loci genotype points to the high variability of the Mpn1 locus. While this locus adds resolution to the five loci scheme, it has been suggested for removal because it is not stable enough (Sun et al., 2013; Chalker et al., 2015).

### 4.6 Association between MLVA subtypes and clinical features

In our cohort, four susceptible strains had no mutations at the common resistance sites. Three of them were M4-5-7-3, a subtype that earlier studies found to be more macrolide-susceptible (Lu et al., 2020; Charlotte Hsiung et al., 2022). The M3-5-6–2 subtype, on the other hand, has become increasingly resistant in recent years, with some reports putting resistance rates at 95.1% or even 100% (Wang et al., 2022; Jiang et al., 2023). Nearly all M3-5-6–2 isolates in our study were resistant, which matches this trend. As for the dominant M4-5-7-2 genotype, its resistance pattern varies a lot by location and over time. In China, resistance rates for this type have reached 90% or even 99.4% (Wang; Jiang et al., 2023). Yet in the United States, Europe, and Australia, where this genotype is also common, macrolide resistance rates remain comparatively low (Lu et al., 2020; Xiao et al., 2020). Similarly, surveillance data from Japan have shown temporal fluctuations in resistance rates for this type (Lu et al., 2020).

Using the five loci MLVA scheme, we found that subtype M4-3-5-6-2 was protective against SMPP, whereas subtype M3-4-5-7-2 carried the highest risk, followed by subtypes M2-4-5-7-2. These findings align with earlier work conducted in Beijing (2010-2012) suggested that infections with subtype M3-4-5-7–2 were associated with higher Pneumonia Severity Index scores (Qu et al., 2013). While our four loci analysis revealed that the three genotypes identified (M4-5-7-2, M3-5-6-2, and M4-5-7-3) did not show a significant association with disease severity. This finding differs from an earlier report by Yan et al. (2019), who suggested that the M3-5-6-2 type may increase the risk of progression to SMPP (Yan et al., 2019). The discrepancy may be explained by the limited discriminatory power of the four loci scheme compared to the five loci system, which includes the Mpn1 locus and provides finer strain differentiation. These observations underscore the importance of using high-resolution genotyping methods when exploring associations between MLVA subtypes and clinical outcomes. The association between MLVA subtypes and macrolide resistance or the severity of MPP remains debated in China (Qu et al., 2013; Zhu et al., 2026). Nevertheless, our results highlight the potential value of MLVA genotyping for tracking drug-resistance trends and assessing disease severity in children with MPP.

Beyond these epidemiological associations, the potential biological significance of Mpn1 variation deserves further attention. The Mpn1 locus corresponds to the *hsdS* gene *MPN089*, which encodes the specificity subunit *(*HsdS*)* of the type I restriction–modification system. Within the dominant four loci genotype M4-5-7-2, variation at this locus was linked to different risks of SMPP. Shorter repeats (2–3 copies) were more common in severe cases. Although Mpn1 is left out of the standard four loci MLVA scheme because it changes quickly and lacks long-term stability (Sun et al., 2013; Chalker et al., 2015), tandem repeat variation in the *hsdS* gene has been tied to macrolide resistance. Lee et al. (2022) found that resistant strains tended to carry specific *hsdS* repeat patterns at *MPN085* and *MPN285*, while susceptible strains more often had shorter or missing repeats. Notably, the mpn1 gene (*MPN089*), which was dropped from the original MLVA scheme, was also a focus of their work. Their findings suggested that tandem repeat number variation at this locus may be linked to macrolide resistance (Lee et al., 2022). Wang et al. (2023) reported a 12-bp tandem repeat insertion in another *hsdS* gene, *MPN365*, in a roxithromycin-induced resistant mutant—the first time *hsdS* variation was seen alongside a change in macrolide susceptibility (Wang et al., 2023). Exactly how the *hsdS* gene contributes to resistance is still unclear. Whether Mpn1 repeat variation directly affects virulence, alters host interactions, or simply tracks with genotypes linked to severe disease remains unknown. Even so, our finding that specific Mpn1 repeat variants within the M4-5-7–2 background were tied to SMPP risk suggests that Mpn1 variability may matter beyond strain typing and deserves further study.

## 5 Conclusion

This study systematically investigated the epidemiological, molecular, and clinical characteristics of pediatric MPP in Liaocheng, China, during 2023–2024. Our findings reveal an extremely high genotypic macrolide resistance rate of 97.30%, predominantly attributed to the A2063G point mutation in domain V of the 23S rRNA gene. Elevated serum levels of LDH and D-dimer were identified as independent risk factors for SMPP, supporting their potential utility as early biomarkers for evaluating disease severity in children with MPP.

MLVA genotyping further demonstrated distinct associations between strain subtypes and clinical severity. Subtype M4-3-5-6–2 appeared to confer protection against severe disease, whereas subtype M3-4-5-7–2 was strongly linked to increased SMPP risk. These results suggest that MLVA typing can provide additional prognostic insights that complement conventional resistance detection, helping refine individual risk stratification.

Despite widespread macrolide resistance, azithromycin remained clinically effective in most pediatric patients. This underscores the importance of personalized therapeutic strategies tailored to early disease assessment and dynamic clinical monitoring.

Collectively, this study highlights the clinical value of integrating molecular surveillance, including resistance mutation screening and MLVA genotyping, with routine clinical and laboratory evaluation. Such a combined approach can substantially optimize clinical decision-making and improve prognostic management for pediatric MPP.

## 6 Study limitations

This study has several limitations. First, the retrospective case-control design and targeted patient enrollment may limit the generalizability of prevalence estimates to broader populations. Second, MLVA genotyping was performed on only 126 of the 685 PCR-positive specimens, as DNA degradation in the remaining samples precluded reliable genetic analysis. Third, macrolide resistance was assessed solely by detecting known resistance-associated mutations, without confirmatory phenotypic susceptibility testing. Finally, the single-center retrospective setting and inpatient-predominant cohort may restrict the applicability of our findings to outpatient or community-based populations. Therefore, further multicenter prospective studies are warranted to validate and extend these results.

## Data Availability

The datasets presented in this study can be found in online repositories. The names of the repository/repositories and accession number(s) can be found in the article/supplementary material.
